# Omicron SARS-CoV-2 shows NRP1-linked susceptibility in adipose-derived MSCs and alters primitive cord blood CD34⁺ HSPCs

**DOI:** 10.1371/journal.pone.0360439

**Published:** 2026-10-12

**Authors:** Kanit Bhukhai, Nopmullee Tanhuad, Pornprapa Srimorkun, Suwimon Manopwisedjaroen, Wittaya Panvongsa, Vimvara Vacharathit, Phongthon Kanjanasirirat, Umnuaychoke Thongsa-ad, Supasek Kongsomros, Nareerat Sutjarit, Chanya Srisaowakarn, Tulyapruek Tawonsawatruk, Patompon Wongtrakoongate, Nittaya Boonmuen, Suparerk Borwornpinyo, Usanarat Anurathapan, Arthit Chairoungdua, Nithi Asavapanumas, Suradej Hongeng, Arunee Thitithanyanont

**Affiliations:** 1 Department of Physiology, Faculty of Science, Mahidol University, Bangkok, Thailand; 2 Department of Microbiology, Faculty of Science, Mahidol University, Thailand; 3 Department of Tropical Nutrition and Food Science, Faculty of Tropical Medicine, Mahidol University, Thailand; 4 Single-cell Omics and Systems Biology of Diseases (scSyBiD) Research Unit, Faculty of Science, Mahidol University, Bangkok, Thailand; 5 Department of Pathobiology, Faculty of Science, Mahidol University, Bangkok, Thailand; 6 Graduate Program in Nutrition, Faculty of Medicine Ramathibodi Hospital, Mahidol University, Thailand; 7 Department of Orthopaedics, Faculty of Medicine, Ramathibodi Hospital, Mahidol University, Bangkok, Thailand; 8 Department of Biochemistry, Faculty of Science, Mahidol University, Bangkok, Thailand; 9 Department of Biotechnology, Faculty of Science, Mahidol University, Bangkok, Thailand; 10 Department of Pediatrics, Faculty of Medicine, Ramathibodi Hospital, Mahidol University, Bangkok, Thailand; 11 Chakri Naruebodindra Medical Institute, Faculty of Medicine Ramathibodi Hospital, Mahidol University, Samut Prakan, Thailand; Fudan University, CHINA

## Abstract

SARS-CoV-2 infection is linked to hematologic abnormalities, but whether early hematopoietic compartments and their stromal niche are directly engaged remains unclear. We used in vitro exposure models to compare variant-associated responses of adult mesenchymal stromal cells (MSCs) and umbilical cord blood (UCB) CD34 ⁺ hematopoietic stem and progenitor cells (HSPCs). Adipose–derived (AD) and bone marrow–derived (BM) MSCs exposed to wild-type, Delta, or Omicron showed source- and variant-dependent nucleocapsid protein (NP) positivity, highest with Omicron in AD-MSCs; infectious virus in supernatants was undetectable. AD-MSCs expressed higher neuropilin-1 (NRP1) than BM-MSCs, and NRP1 knockdown reduced Omicron-associated NP signal, implicating an NRP1-linked entry process. SARS-CoV-2 exposure induced indoleamine-2,3-dioxygenase 1 (*IDO1*), most prominently in AD-MSCs. In UCB CD34 ⁺ HSPCs, Omicron increased NP positivity enriched in long-term HSCs, reduced primitive fraction frequencies, increased colony formation, and promoted early erythroid progenitor accumulation; heat-inactivated virus did not. NanoString profiling revealed host-response activation and altered cell-cycle/differentiation programs. These data indicate Omicron can engage stromal and early hematopoietic compartments and remodel stress-responsive hematopoiesis.

## Introduction

SARS-CoV-2 infection is typically initiated in the upper respiratory tract, where airway epithelial cells are among the earliest and most prominently infected cell types; however, COVID-19 is a systemic illness with well-documented effects beyond the lungs, including in the hematopoietic and immune compartments [1]. Clinically, severe cases of COVID-19 have been associated with a wide range of hematological abnormalities (e.g., leukopenia, thrombocytopenia, and coagulopathies) alongside dysregulated inflammation [2], raising the possibility that hematopoietic homeostasis is disrupted during acute and post-acute SARS-CoV-2 infection [3]. Although substantial work has focused on interactions between SARS-CoV-2 and mature immune cells and on inflammatory pathways that shape disease severity [4,5] whether early hematopoietic and stromal compartments can be directly altered by SARS-CoV-2 exposure remains unclear.

Hematopoiesis is sustained by hematopoietic stem and progenitor cells (HSPCs), including hematopoietic stem cells (HSCs), which generate all blood and immune lineages throughout life. HSPCs are highly responsive to inflammatory cues and pathogen-sensing pathways, and such signals can shift proliferation, survival, and lineage commitment [6]. These properties make early hematopoietic compartments plausible targets of SARS-CoV-2–associated perturbation, either through direct viral interactions or through indirect signaling mechanisms, with potentially broad downstream consequences for immune competence and hematologic recovery.

Early hematopoietic behavior is also shaped by the stromal niche, which provides critical trophic and regulatory signals. Mesenchymal stromal cells (MSCs) are key stem cell niche components with immunomodulatory capacity [7] and recognized clinical relevance, having been investigated as potential cell-based therapies for COVID-19–associated hyperinflammation [8]. Importantly, MSCs are heterogeneous across tissue sources; bone marrow–derived MSCs (BM-MSCs) and adipose-derived MSCs (AD-MSCs) are among the most commonly used adult MSC sources in translational settings and can differ in phenotype and functional programs [9,10], raising the possibility that their susceptibility to SARS-CoV-2 exposure and their downstream niche responses may also vary by source.

Viral evolution further motivates direct evaluation of these compartments. SARS-CoV-2 variants differ in transmissibility, immune escape, and entry properties, and Omicron lineages in particular display altered cellular tropism relative to earlier strains [11–14]. In addition to canonical entry determinants, accessory factors can influence viral entry in cell types with low Angiotensin-Converting Enzyme 2 (ACE2)/Transmembrane Serine Protease 2 (TMPRSS2) expression. For instance, Neuropilin-1 (NRP1) has been implicated as a facilitator of SARS-CoV-2 entry in certain cellular contexts and may be especially relevant in stromal cells, where canonical receptor expression can be limited [15]. Whether variant-associated differences extend to MSCs from distinct tissue sources and to primitive HSPC compartments, and whether factors such as NRP1 contribute to these differences, remains unclear.

Here, we investigated SARS-CoV-2 variant-associated effects in adult MSCs and umbilical cord blood (UCB) CD34 ⁺ HSPCs using *in vitro* exposure models. AD-MSCs and BM-MSCs were exposed to Wuhan wild-type, Delta, and Omicron (BA.1.15) to assess nucleocapsid protein (NP) positivity, infectious virus release in culture supernatants, and induction of the immunomodulatory marker indoleamine-2,3-dioxygenase 1 (*IDO1*). To probe potential entry determinants, we profiled entry-associated factor expression and tested the contribution of NRP1 to Omicron-associated NP positivity in AD-MSCs using siRNA-mediated knockdown. In parallel, we evaluated Omicron-associated NP positivity across immunophenotypically defined HSPC compartments, including a long-term HSC (LT-HSC) phenotype subset, and assessed downstream functional and developmental outcomes using colony-forming assays, erythroid progenitor profiling, and targeted NanoString transcriptional analysis. Together, these studies aim to define variant- and cell-type–associated susceptibility patterns and characterize stromal and hematopoietic remodeling linked to SARS-CoV-2 exposure.

## Materials and methods

### Ethics Statement

UCB was obtained from the National Blood Centre, Thai Red Cross Society, under the approval of Research Ethics Committee, the National Blood Centre, Thai Red Cross Society (COA No. NBC 6/2022) after receiving informed written consent from the participants between 27/04/2022 and 27/04/2023. The study and all methods were performed in accordance with the protocols approved by the Human Research Ethics Committee, Faculty of Medicine Ramathibodi Hospital, Mahidol University (COA No. MURA 2022/65).

### Isolation and culture of UCB-CD34^+^ HSPCs

Human umbilical cord blood mononuclear cells (UCB-MNCs) were isolated by density gradient centrifugation (Lymphoprep, STEMCELL Technologies). CD34^+^ cells were isolated from UCB-MNCs using EasySep human CD34 positive selection kit (STEMCELL Technologies) according to manufacturer’s protocol. The efficiency of UCB-CD34^+^ HSPCs was determined by flow cytometry and ranging from > 95%. The CD34 positive cells were cultured in StemSpan™-AOF expansion medium (STEMCELL Technologies) supplemented with 150 ng/mL stem cell factor (SCF), 150 ng/mL thrombopoietin (TPO), 150 ng/mL FLT-3 ligand (Flt-3 L), 10 ng/mL interleukin-6 (IL-6), (all from Peprotech, US). The culture was incubated 37°C in a humidified incubator with 5% CO2.

### MSC and VeroE6 cell cultivation

Human AD-MSCs and BM-MSCs were obtained from the American Type Culture Collection (ATCC). MSCs were cultured in Dulbecco’s modified Eagle’s medium (DMEM, Hyclone) supplemented with 10% fetal bovine serum (FBS, Hyclone), 1% penicillin/streptomycin (Gibco), and 1% GlutamaxTM (Gibco) with medium changed every 3 days. Once the cells reached 90% confluency, they were trypsinized and passaged for further expansion. VeroE6 cells expressing the transmembrane serine protease TMPRSS2 (Vero-E6/TMPRSS2 cells), derived from African green monkey (Cercopithecus aethiops) kidney epithelial cells, were obtained from NIBIOHN. These cells were cultured in DMEM supplemented with 10% FBS and 1 mg/mL G418. The human airway epithelial cell line Calu-3, obtained from the ATCC, were maintained in DMEM: Nutrient Mixture F-12 (DMEM/F-12, Gibco) supplemented with 10% FBS and 1% penicillin/streptomycin. All cell cultures were grown at 37°C in a humidified incubator with 5% CO2.

### SARS-CoV-2 infection

Different SARS-CoV-2 variants, SARS-CoV-2/01/human/ THA/Jan2020 (Wuhan wild-type), SARS-CoV-2/human/THA/2021 (Delta), and SARS-CoV-2/human/THA/2022 (Omicron BA.1.15), were propagated in Vero E6/TMPRSS2 cells using our established methods. All experiments involving live SARS-CoV-2 viruses were conducted within a certified biosafety level 3 (BSL-3) facility at the Department of Microbiology, Faculty of Science, Mahidol University. Prior to infection, MSCs or UCB-CD34^+^ HSPCs were plated one day in advance. The cells were then rinsed with serum-free media and allowed to adsorb the virus for 2 hours, using 50% of tissue culture infectious dose (TCID50) for each virus variant. Subsequently, the cells were washed three times with serum-free growth media and maintained in serum-free media for 72 hours.

### Immunophenotyping of human HSPCs

To quantify HSPC subsets, cells were collected before and after SARS-CoV-2 exposure and fixed in 4% paraformaldehyde (PFA). Fixed cells were washed twice with 1X DPBS and stained with fluorophore-conjugated antibodies against human HSPC surface markers (PerCP anti-human CD34 antibody, PE anti-human CD38 antibody, Brilliant Violet 510™ anti-human CD90 (Thy1) antibody, and Brilliant Violet 421™ anti-human CD49f antibody, all from BioLegend). Flow cytometric analysis was performed using the BD AccuriTM C6 (BD Biosciences) and data were analyzed using FlowJo software.

### Colony-forming unit assays

Seventy-two hours after SARS-CoV-2 infection, infected UCB-CD34^+^ HSPCs were plated at a density of 1,000 cells with Methocult^TM^ H4434 (Stemcell technologies) in 6-well plates in duplicate. After 14 days of culture, the colonies were distinguished into erythroid burst-forming units (BFU-E) and granulocyte-macrophage colony-forming units (CFU-GM). Colonies were counted under an inverted microscope.

### Virus inhibition assay

The effects of SARS-CoV-2 infection on UCB-CD34^+^ HSPCs were evaluated using a virus inhibition assay. SARS-CoV-2 wild-type and Omicron variant were inactivated by incubation in a pre-warmed dry heat block at 56 °C for 30 minutes. UCB-CD34^+^ HSPCs were subsequently exposed to either live or heat-inactivated virus to assess SARS-CoV-2 nucleoprotein expression, HSPC immunophenotype, and *in vitro* differentiation.

### High-content imaging system for SARS-CoV2 nucleoprotein detection

The cells were cultured in a 96-well black-plate and fixed using 4% paraformaldehyde in phosphate-buffered saline (PBS). To allow permeabilization, 0.1% Triton-X in PBS was applied to the cells for 10 min. Subsequently, the cells were washed with PBS containing 0.05% Tween detergent (PBST) and then blocked using PBS with 2% (w/v) BSA for 1 hour at room temperature. Following the blocking step, a primary rabbit monoclonal antibody specific to the SARS-CoV Nucleoprotein (NP) (Sino Biological Inc.) was added to the cells at a 1:500 dilution ratio and incubated for 1 hour at 25°C. To remove any unbound antibodies, the cells were washed three times with PBST. A secondary antibody, goat anti-rabbit IgG Alexa Fluor 488 from Thermo Fisher Scientific, was then added at a 1:500 dilution ratio. The nuclei of the cells were stained using Hoechst dye obtained from Thermo Fisher Scientific. The Operetta high-content imaging system (PerkinElmer) was used to detect and analyze the fluorescent signals. Automated quantification of infected cells was performed using the Harmony software (PerkinElmer), which was set to analyze 13 images per well.

### Flow cytometry for determination of viral infection

At 72 hours post-infection, cells were trypsinized and fixed with 4% paraformaldehyde in PBS. Subsequently, the cells were permeabilized using 0.1% Triton-X in PBS. To stain the viral NP, a primary rabbit mAb antibody specific for SARS-CoV NP (Sino Biological Inc.) was used at a dilution ratio of 1:1000. As a secondary antibody, goat anti-rabbit IgG Alexa Fluor 488 (Thermo Fisher Scientific) was employed at a dilution ratio of 1:1000. The cells were then washed and resuspended in PBS before being subjected to flow cytometric analysis using the BD AccuriTM C6 (BD Biosciences). Flow cytometric data were analyzed using Flowjo software. For MSCs, NP positive cells were quantified as a percentage of total cells comparing to mock condition (unexposed cells). The NP positive among UCB-CD34^+^ HSPCs was determined as a percentage of total cells and the percentage among HSPC subpopulations.

### Focus-forming assay

Cultured supernatants were collected from SARS-CoV-2-infected MSCs to assess viral output. The viral output in supernatants were determined by a focus-forming assay. In brief, Vero-E6/TMPRSSs cell monolayer was seeded into 96-well plate 24 hours before infection. Cells were inoculated with a serial dilution of the virus-containing supernatants for 1 hour in an incubator at 37°C. Then, the cells were overlaid with 200 uL /well of overlay medium containing DMEM supplemented with 2% FBS and 2% carboxymethyl cellulose (CMC). The culture was incubated at 37 °C in 5% CO2 for 1 day for Wuhan wild-type and Delta variant and 2 days for Omicron variant. After that, the plate was stained with 1:2500 of rabbit anti-SARS-CoV NP (Sino Biological Inc.) and then anti-rabbit HRP (DAKO) was used as secondary antibody. Trueblue substrate (Seracare) was then added and incubated in the dark for 10 min. The FFU per well was quantified using Cytation 7. The infectious viral concentrations are reported as focus-forming units per milliliter (FFU/mL).

### Quantitative real-time PCR

After collecting infected MSCs, the cells were subjected to three washes with PBS. Total RNA was extracted from the cells using the RNeasy minikit (Qiagen) following the manufacturer’s instructions. Subsequently, cDNA was synthesized using the iScript™ Reverse Transcription Supermix for RT-qPCR (Biorad). The expression of mRNA was evaluated using real-time PCR with specific primers and SYBR Green (Bio-Rad) on an ABI PRISM7500 Sequence Detection System, equipped with analytical software from Applied Biosystems (Thermo Fisher Scientific). The primer sequences used were as follows: *IDO1*_forward- 5’ GCCCTTCAAGTGTTTCACCA 3′; *IDO1*_reverse- 5’ TGCCTTTCCAGCCAGACAA 3’; *GAPDH*_forward- 5’ ATGCCCCCATGTTCGTCATG 3′; *GAPDH*_reverse-5’ GCAGGAGGCATTGCTGATGA 3′.

### Western blot analysis

The cells were washed twice with cold phosphate-buffered saline and then lysed using modified radioimmunoprecipitation assay buffer. This buffer contains the following components in millimolar concentrations: 50 Tris-HCl (pH 7.4), 150 NaCl, 1 EDTA, 1 NaF, 1 Na3VO4, 1 PMSF, and 1% Triton X-100. Additionally, the buffer contained a protease inhibitor cocktail and a phosphatase inhibitor. Following sonication and incubation on ice, the samples were centrifuged at 12,000 rpm for 30 minutes at 4 °C. An equal amount of protein was then mixed with 6X Laemmli buffer and heated at 95 °C for 5 minutes. The samples were resolved through sodium dodecyl sulfate polyacrylamide gel electrophoresis and subsequently transferred to an Immobilon®-P membrane (PVDF, Merck) via electroblotting. The membranes were incubated overnight at 4 °C with primary antibodies, rabbit anti-ACE2 (Abcam), rabbit anti-TMPRSS2 (Abcam), mouse anti-NRP1 (Santa Cruz Biotechnology), and mouse anti-β-actin (Merck). After washing the membranes five times with Tris-buffered saline containing Tween 20, they were incubated with HRP-conjugated secondary antibodies. Finally, the signals were detected using Immobilon® Crescendo Western HRP Substrate (Millipore).

### siRNA-mediated knockdown of NRP1

AD-MSCs were transfected with 80 pmol of NRP1 siRNA or non-targeting siRNA, all from Santa Cruz Biotechnology, using Lipofectamine 3000 transfection reagent (Invitrogen), following the manufacturer’s instructions. After 24 hours, the transfected cells were evaluated for NRP1 protein expression and subsequently infected with different SARS-CoV-2 variants.

### NanoString platform analysis for Omicron infected HSPCs profiling, differential expression, and gene set enrichment analysis

Total RNA was isolated from UCB-CD34^+^ HSPCs 72 h after exposure to Omicron SARS-CoV-2 or mock (RNeasy Mini Kit, Qiagen). RNA concentration and integrity were assessed by Nanodrop. 100 ng RNA per sample was hybridized for 12 h at 65 °C to the nCounter Human Stem Cell Characterization or Human Host Response CodeSet and processed on the nCounter (NanoString Technologies). Counts were background-subtracted using the geometric mean of the eight negative control probes, then normalized to the six positive control probes and to the geometric mean of the twelve housekeeping genes supplied with each CodeSet, and log2-transformed. All endogenous genes were retained. Differential expression was assessed using limma with empirical Bayes moderated t-statistics and Benjamini–Hochberg adjustment across all endogenous genes in each panel. No gene reached a 5% false discovery rate; genes were therefore defined as differentially expressed at a nominal *P* < 0.05 with |log2 fold change| > 1, and all transcriptional results are reported as exploratory. Volcano plots used ggplot2 and ggrepel R package. Gene set enrichment analysis used clusterProfiler R package on all endogenous genes ranked by log2 fold change, against MSigDB v2025.1 Gene Ontology biological process sets.

### Statistical analysis

Data from a minimum of three independent experiments with a biological replicate were included in the analysis. For conditions in which fewer than three independent biological replicates, the data were presented descriptively and were not included in inferential statistical comparisons. The Delta condition was represented by two independent biological replicates (Fig. 1D) and was therefore excluded from the statistical analysis. Statistical differences between groups were assessed using appropriate statistical tests, including the paired student’s t-test, unpaired student’s t-test, one-way analysis of variance. The analyses were performed using GraphPad Prism version 8.0 for Windows (GraphPad Software). *P* < 0.05 indicated statistical significance.

**Fig 1:**
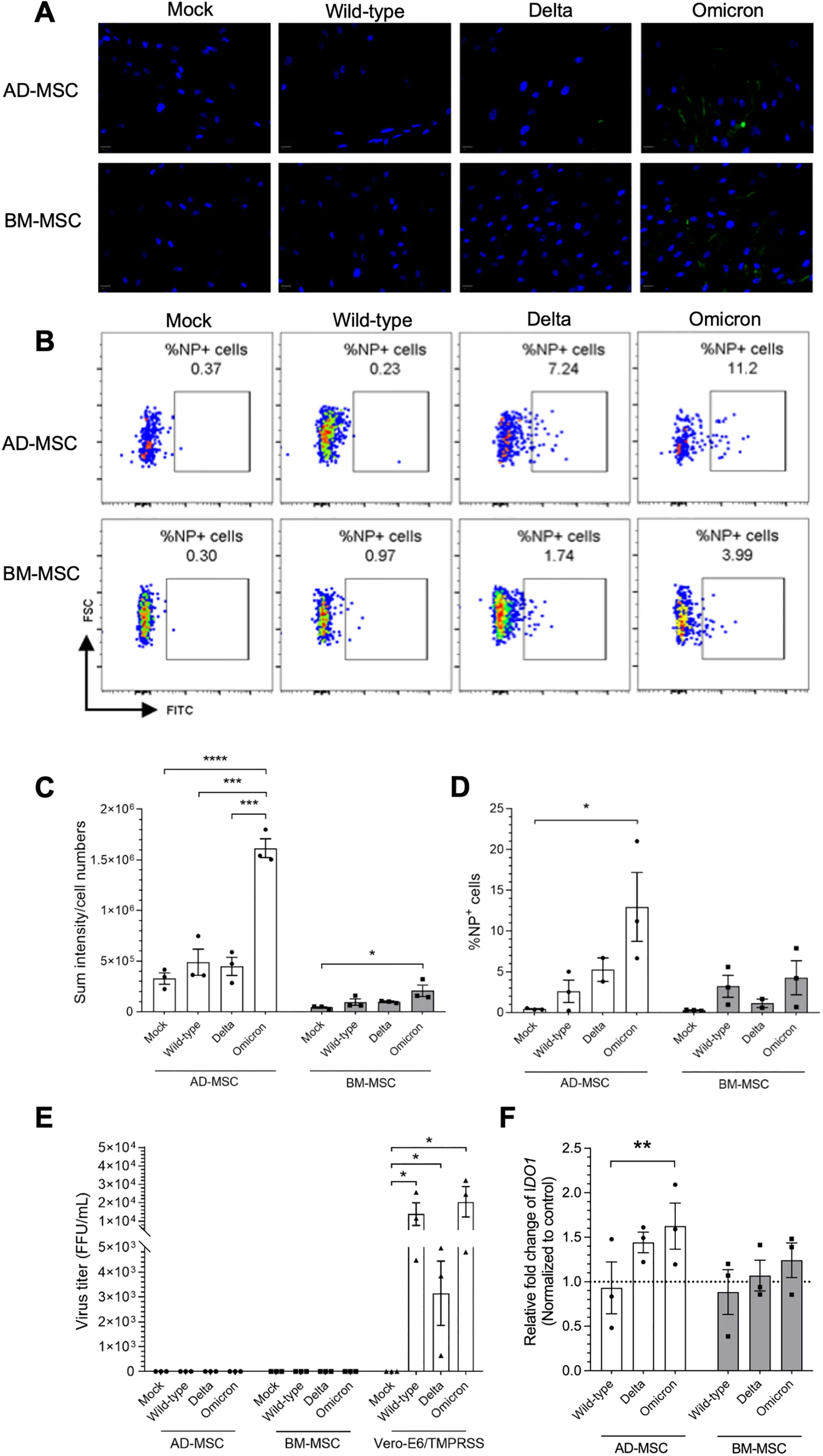
Variant- and tissue source-dependent SARS-CoV-2 NP positivity and *IDO1* induction in MSCs without detectable infectious virus release. **(A)** Detection of SARS-CoV-2 infected cells using high-content imaging under control conditions. Fluorescent signals: green (anti-SARS-CoV NP mAb), blue (Hoechst). **(B)** Representative flow cytometry analysis plots showing NP+ cells. **(C)** Measurement of fluorescent intensity in NP+ cells. **(D)** Percentage of infected MSCs. **(E)** The supernatants of infected MSCs were collected and titrated on Vero-E6/TMPRSS2 cells to measure infectious viral particle production using the focus-forming assay. **(F)** After RNA extraction from infected MSCs, the expression of *IDO1* was examined using real-time PCR. The graph displays the expression of *IDO1* normalized to *GAPDH*, with the mock group serving as the control. Data are represented as means ± SEM from three independent biological replicates. For Fig 1D, the Delta condition was available from only two independent donors and was therefore presented descriptively and excluded from the statistical analysis. Statistical comparisons among the remaining groups were performed using one-way ANOVA followed by Tukey’s multiple-comparisons test. One-way ANOVA showed a significant overall difference among groups, F(2, 6) = 6.794, *P* = 0.0287. * *P*  <  0.05, ** P  <  0.01, *** *P*  =  0.0001, **** *P*  <  0.0001.

## Results

### MSCs show variant- and tissue source-dependent SARS-CoV-2 nucleocapsid positivity and *IDO1* induction without detectable infectious virus release

To evaluate the susceptibility of MSCs to SARS-CoV-2 infection, AD- and BM-MSCs were infected with three variants, namely Wuhan wild-type (WT), Delta, and Omicron. The presence of viral NP was examined using immunofluorescent (IF) staining and flow cytometry analysis in MSCs at 72-hour post-infection (hpi). For IF staining, the level of NP-positivity was demonstrated as sum intensity/cell numbers. AD-MSCs exhibited a significantly higher level of NP-positivity for Omicron infection compared to WT (*P* = 0.0001) and Delta (*P* = 0.0001) variant for approximately 3-fold. BM-MSCs showed a slightly significant increase in NP-positivity compared to Mock group after infected with Omicron variant (*P* = 0.0322), but the expression of NP in BM-MSC did not exhibit significant difference compared to WT and Delta variant (Fig 1A and 1C). After that, flow cytometry was employed to assess the percentage of NP+ cells. Consistent with IF analysis, Omicron-infected AD-MSCs demonstrated significantly higher percentages of NP+ cells (*P* = 0.0316), although the percentage of NP+ cells in Omicron-infected BM-MSCs showed a slight increase compared to the Mock control but not statistically significant (Fig 1B and 1D). However, for the analysis shown in Fig 1D, three independent biological replicates were available for the mock, WT, and Omicron groups, whereas the Delta group included only two independent donors. Because of the limited number of biological replicates in the Delta group, these data are shown descriptively and were not included in the inferential statistical analysis. To further determine whether SAR-CoV2-exposed MSC produced infectious progeny virus, the infectious viral particles were measured in culture supernatants. Interestingly, no infectious virus was detected by FFA in supernatants from WT-, Delta-, or Omicron- exposed AD-MSCs or BM-MSCs at 72 hpi (Fig 1E). Finally, to test whether SARS-CoV-2 exposure elicits functional immunomodulatory responses in MSCs, we quantified Indoleamine-2,3-dioxygenase 1 (*IDO1*) mRNA by RT–qPCR at 72 hpi. Our results demonstrated that *IDO-1* expression was upregulated in AD-MSCs infected with Delta and Omicron variants. Moreover, the upregulation was statistically significant in Omicron-infected AD-MSCs compared to the WT (*P* = 0.0094) (Fig 1F). Together, these data indicate that adult MSCs, particularly AD-MSCs, exhibit increased Omicron-associated NP positivity and activation of an *IDO1*-associated immunomodulatory program, while not releasing detectable infectious virus under the conditions tested.

### AD-MSCs and BM-MSCs differ in expression of SARS-CoV-2 entry-associated factors

To assess whether differences in entry-associated factors might contribute to MSC source-dependent NP positivity, we measured ACE2, TMPRSS2, and NRP1 protein levels in AD-MSCs and BM-MSCs by Western blot (with Calu-3 as a positive control). Interestingly, the expression level of ACE2 and TMPRSS2 detected on AD-MSCs and BM-MSCs were markedly low comparing to Calu-3 cells, whereas the expression of NRP1 in AD-MSC was higher compared to BM-MSC (*P* = 0.0110) and Calu-3 cells (*P* = 0.0409) (Fig 2A-2C; **S1 Raw images**). These findings raise the possibility that NRP1 may be involved in enhancing SARS-CoV-2 infection in AD-MSCs when canonical entry machinery is comparatively limited.

**Fig 2:**
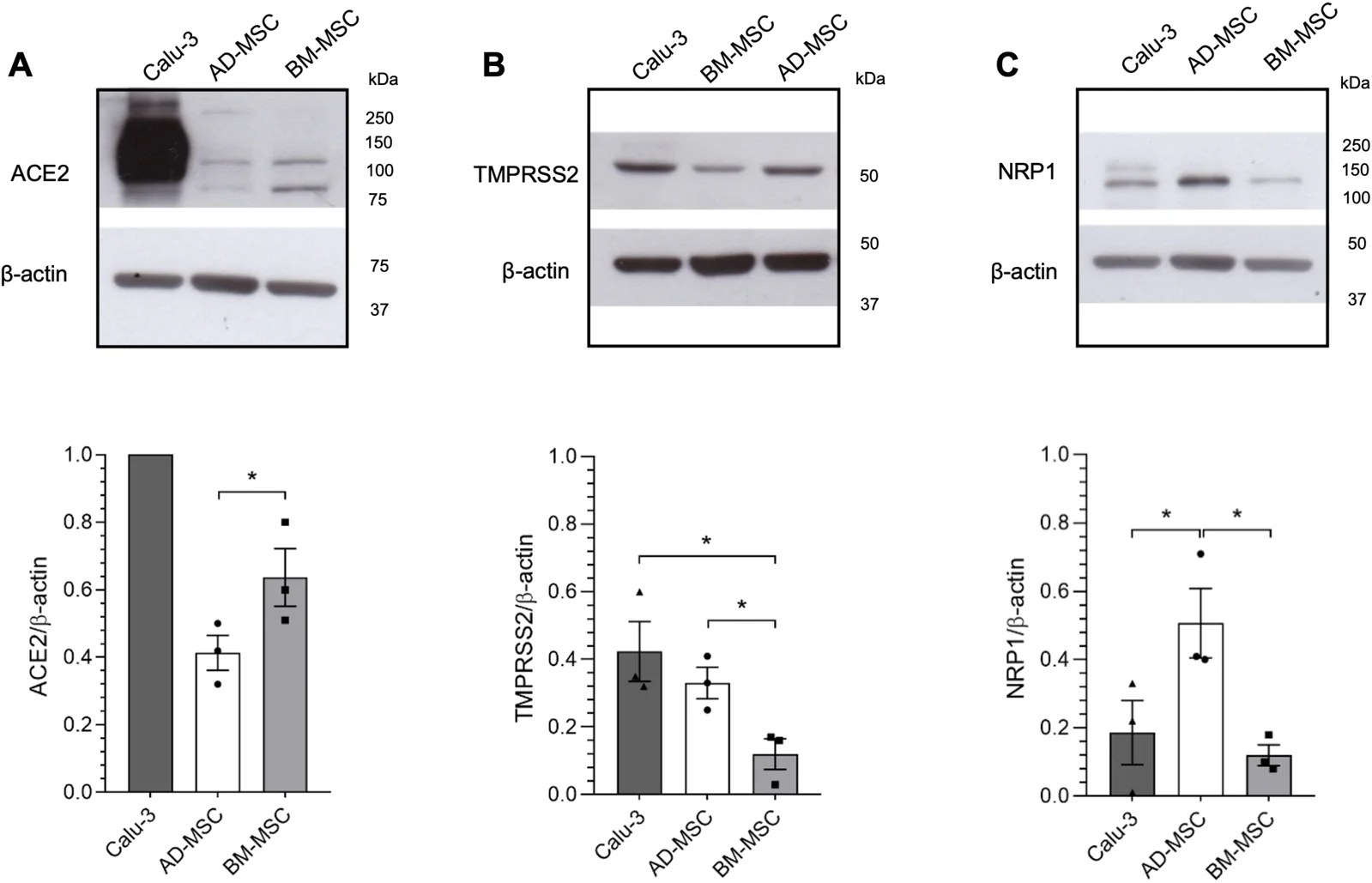
Expression of ACE2, TMPRSS2, and NRP1 in adipose- and bone marrow-derived MSCs. The expression of ACE2, TMPRSS2, and NRP1 in human MSCs derived from adipose tissue (AD-MSC) and bone marrow (BM-MSC) was examined. A representative Western blot of ACE2 **(A)**, TMPRSS2 **(B)**, and NRP1 **(C)** is shown on the upper side. The quantification of ACE2 **(A)**, TMPRSS2 **(B)**, and NRP1 **(C)** is presented in the graphs on the lower side. Calu-3 cells were used as a positive control cell line. β-actin was used as the loading control for cell lysates. The data are represented as mean ± SEM from three independent biological replicates. Statistical comparisons were performed using an unpaired t-test between AD-MSCs and BM-MSCs. * *P*  <  0.05, ** P  <  0.01, *** *P*  =  0.0001, **** *P*  <  0.0001.

### siRNA-mediated knockdown of NRP1 selectively reduces Omicron-associated NP signal in AD-MSCs

Related studies have reported that NRP1 enhances SARS-CoV-2 infection when co-expressed with ACE2 and/or TMPRSS2 [15–17]. We further investigated the role of NRP1 in the entry and infectivity of SARS-CoV-2-infected AD-MSCs using siRNA knockdown technique. AD-MSCs were transfected with siRNA targeting NRP1 (siNRP1) or control siRNA (siControl). After 24 hours of transfection, the expression of NRP1 was assessed through immunoblotting (Fig 3A and 3B; S1 Raw images) and immunofluorescence staining (Fig 3C and 3D), which confirmed significant attenuation of NRP1 expression in siNRP1-transfected AD-MSCs (immunoblotting: siControl vs. siNRP1 *P* = < 0.0001, IF siControl vs. siNRP1 *P* = 0.0008). Subsequently, transfected AD-MSCs were infected with different SARS-CoV-2 variants (Wild-type, Delta, and Omicron). At 72 hpi, the susceptibility of AD-MSCs to infection was determined using immunofluorescence staining (Fig 3E and 3F). Of note, the siRNA-mediated knockdown of NRP1 significantly reduce the Omicron-associated NP signal in AD-MSCs (*P* = 0.0431), while WT and Delta NP signals were not meaningfully altered under the same conditions (Fig 3E and 3F). These results indicate that NRP1 contributes to Omicron-associated NP positivity in AD-MSCs suggesting the potential role for NRP1 in facilitating SARS-CoV-2 Omicron entering AD-MSCs.

**Fig 3:**
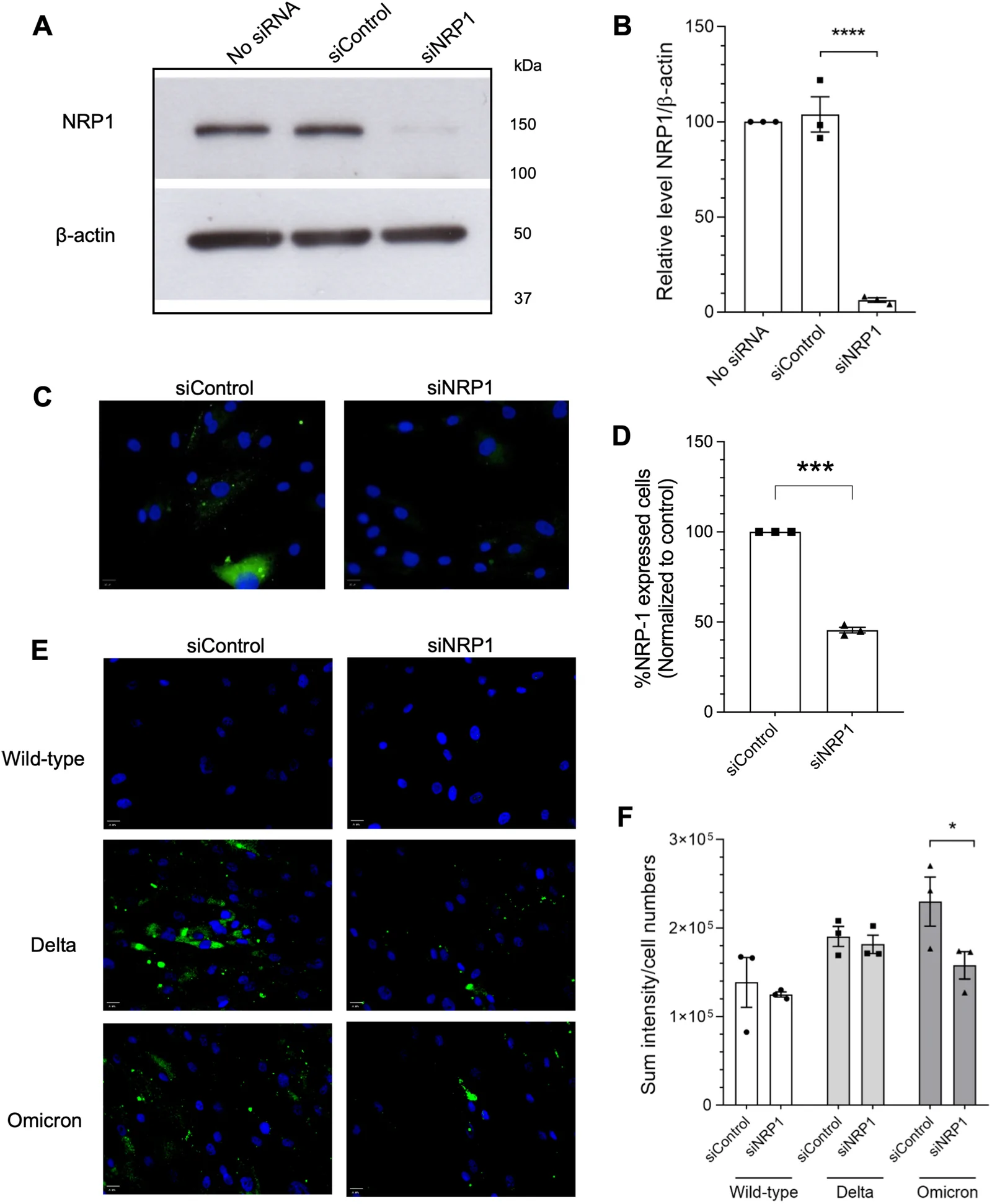
NRP1 knockdown reduces Omicron-associated NP signal in adipose-derived MSCs. **(A)** Representative western blot depicting the expression of NRP1 in AD-MSCs (No siRNA), cells transfected with non-targeting siRNA (siControl), or siNRP1 for 24 hours. β-actin was used as a loading control. **(B)** Graph displaying the quantification of NRP1 normalized to β-actin (n = 3). This experiment represents three independent biological replications. Statistical analysis was performed using one-way ANOVA followed by Tukey’s multiple comparisons test. **(C)** Transfected cells were visualized using high-content imaging. Fluorescent signals: green for anti-NRP1 mAb, blue for Hoechst. **(D)** Percentage of NRP1-expressing cells normalized to cells transfected with siControl. Data are presented as mean ± SEM from three independent experiments. Statistical analysis was conducted using a paired t-test. Next, siRNA-transfected cells were infected with different variants of SARS-CoV-2. **(E)** SARS-CoV-2-infected cells were detected using high-content imaging. Fluorescent signals: green for anti-SARS-CoV NP mAb, blue for Hoechst. **(F)** The fluorescent intensity of NP+ cells was measured. All data represent three independent experiments. Statistical analysis among each group of variant was performed using an unpaired t-test. * *P*  <  0.05, ** P  <  0.01, *** *P*  =  0.0001, **** *P*  <  0.0001.

### Omicron-associated NP positivity and remodeling of primitive UCB CD34 ⁺ HSPC fractions without detectable infectious virus release

To determine whether early hematopoietic compartments exhibit SARS-CoV-2-associated antigen positivity and remodeling, UCB-CD34^+^ HSPCs were exposed to WT or Omicron and analyzed for NP signal across progressively primitive immunophenotypic compartments. NP positivity increased significantly after exposure with Omicron, compared to Mock and WT, showing the strongest NP signal and the greatest enrichment of NP⁺ cells within primitive subsets, including the CD34^+^CD38^−^ (Mock vs. Omicron *P* = 0.0153), CD34^+^CD38^–^CD90^+^ (Mock vs. Omicron *P* = 0.0198, WT vs. Omicron *P* = 0.0298), and LT-HSC-phenotype (CD34 ⁺ CD38 ^−^ CD90 ⁺ CD49f⁺) subsets (Mock vs. Omicron *P* = 0.0129, WT vs. Omicron *P* = 0.0347) (Fig 4A). In parallel, infectious virus in UCB CD34^+^ HSPCs culture supernatants was assessed by focus-forming assay. Infectious virus was detected in permissive control cultures, but was undetectable in UCB CD34^+^ HSPCs supernatants under the conditions tested (Fig 4B). Despite the lack of detectable infectious virus release, WT– and more prominently Omicron– was associated with reduced frequencies of primitive (CD34 ⁺ CD38 ^−^ CD90⁺) and LT-HSC (CD34 ⁺ CD38 ^−^ CD90 ⁺ CD49f⁺) phenotypes (Mock vs. WT *P* = 0.0362, Mock vs. Omicron *P* = 0.0100) (Fig 4C–Fig 4D).

**Fig 4:**
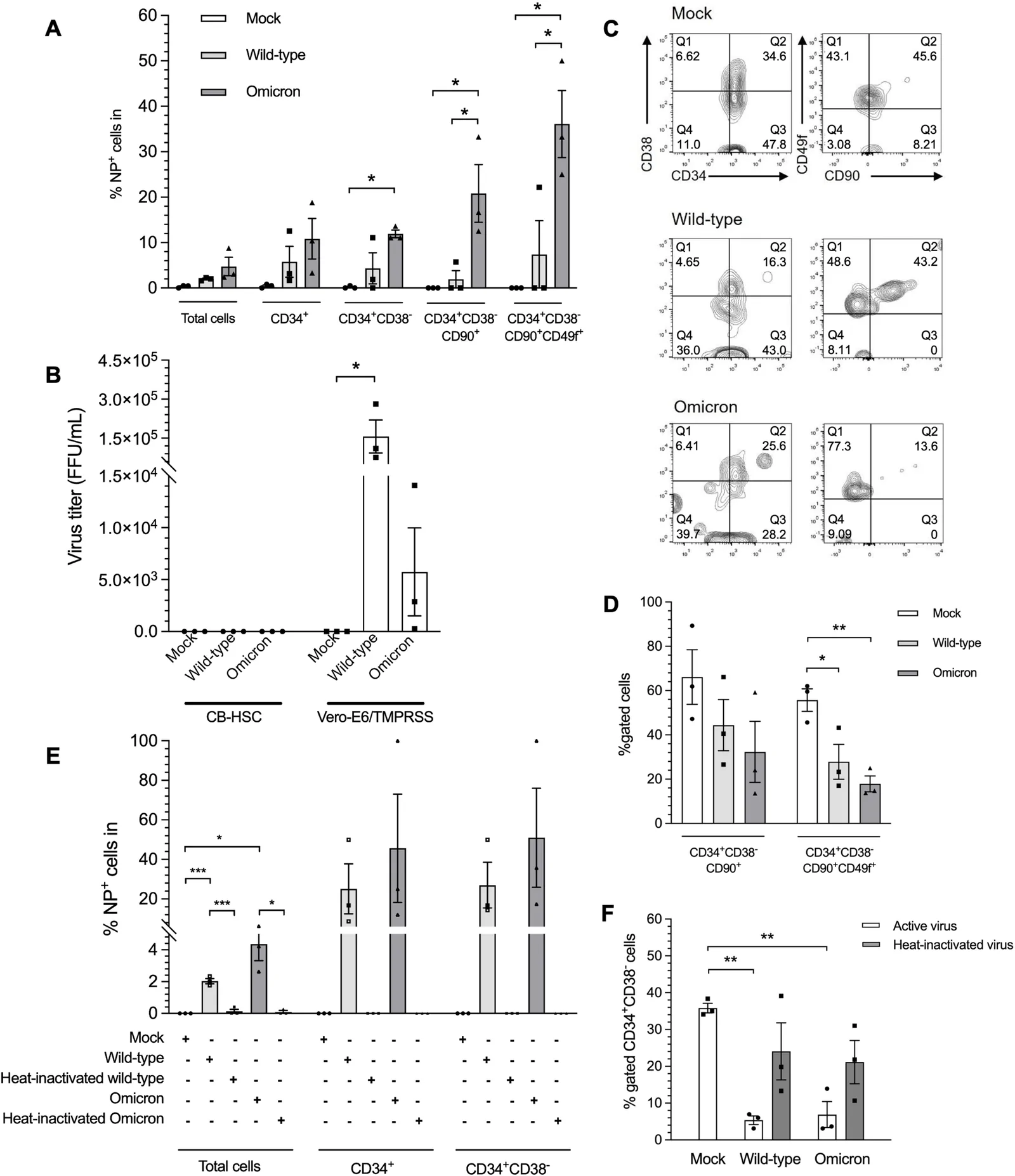
Omicron-associated NP positivity and primitive compartment remodeling in UCB CD34 ⁺ HSPCs, with no detectable infectious virus release. **(A)** Fluorescence intensity of NP-positive UCB-CD34^+^ HSPCs after infection with WT or Omicron variants of SARS-CoV-2. **(B)** Infectious viral particle production was determined by the focus-forming assay on Vero-E6/TMPRSS2 cells. **(C)** HSPC phenotype after infected with SARS-CoV-2 was investigated via the expression of primitive markers through flow cytometry. **(D)** The representative of HSC phenotype after infected with SARS-CoV-2. **(E)** Fluorescent intensity of NP-positive UCB-CD34^+^ HSPCs after infected with heat-inactivated WT and Omicron variant of SARS-CoV-2 compared to active virus. **(F)** The representative of HSC phenotype after infected with heat-inactivated WT and Omicron compared to active virus. All data are represented as mean ± SEM from three independent biological replicates. Statistical comparisons among Mock, WT, and Omicron in each group were performed using one-way ANOVA followed by Tukey’s multiple-comparisons test. For Fig 4E, the unpaired t-test were performed among each group by comparing between Mock vs. WT, Mock vs. Omicron, WT vs. heat-inactivated WT, and Omicron vs. heat-inactivated Omicron. * *P*  <  0.05, ** P  <  0.01, *** *P*  =  0.0001, **** *P*  <  0.0001.

To further assess these effects, we compared UCB CD34^+^ HSPCs exposed to non-heat-inactivated virus with those exposed to heat-inactivated viral material. The heat-inactivated virus did not produce detectable NP signal and did not reproduce the reductions in key primitive fractions observed with non-heat-inactivated virus (Fig 4E–4F). Collectively, these findings indicate that Omicron exposure is associated with preferential NP detection in primitive HSPC compartments and remodeling of primitive phenotype frequencies, occurring without detectable infectious virus release.

### SARS-CoV-2 Omicron variant infection altered hematopoietic differentiation with the accumulation of erythroid progenitors

Because phenotypic shifts in stem/progenitor fractions do not necessarily predict functional output, we performed CFU assays to quantify the multilineage differentiation of virus-exposed UCB CD34 ⁺ HSPCs. WT exposure showed a modest, non-significant trend toward increased CFU output compared with mock, whereas Omicron exposure significantly increased total CFUs (*P* = 0.0021), with contributions from both BFU-E (erythroid) (*P* = 0.0374) and CFU-GM (granulocyte-macrophage) colonies (*P* = 0.0022). (Fig 5A–5B). Next, we used flow cytometry to assess erythroid progenitor composition. At day 14 post-exposure, early erythroid progenitors (CD34^–^CD36^+^CD71^+^CD235^–^ cells) were increased in the culture of SARS-CoV-2-exposed HSPCs, most prominently in the Omicron condition (*P* = 0.0299) (Fig 5C). Virus inhibition experiments supported dependence on exposure to active virus: heat-inactivated virus did not reproduce the increased CFU output (Fig 5D) or the early erythroid progenitor accumulation seen with active virus (Fig 5E). These results indicate that SARS-CoV-2 Omicron exposure can reshape *in vitro* hematopoietic outputs, including erythroid developmental patterns, in an active virus-dependent manner.

**Fig 5:**
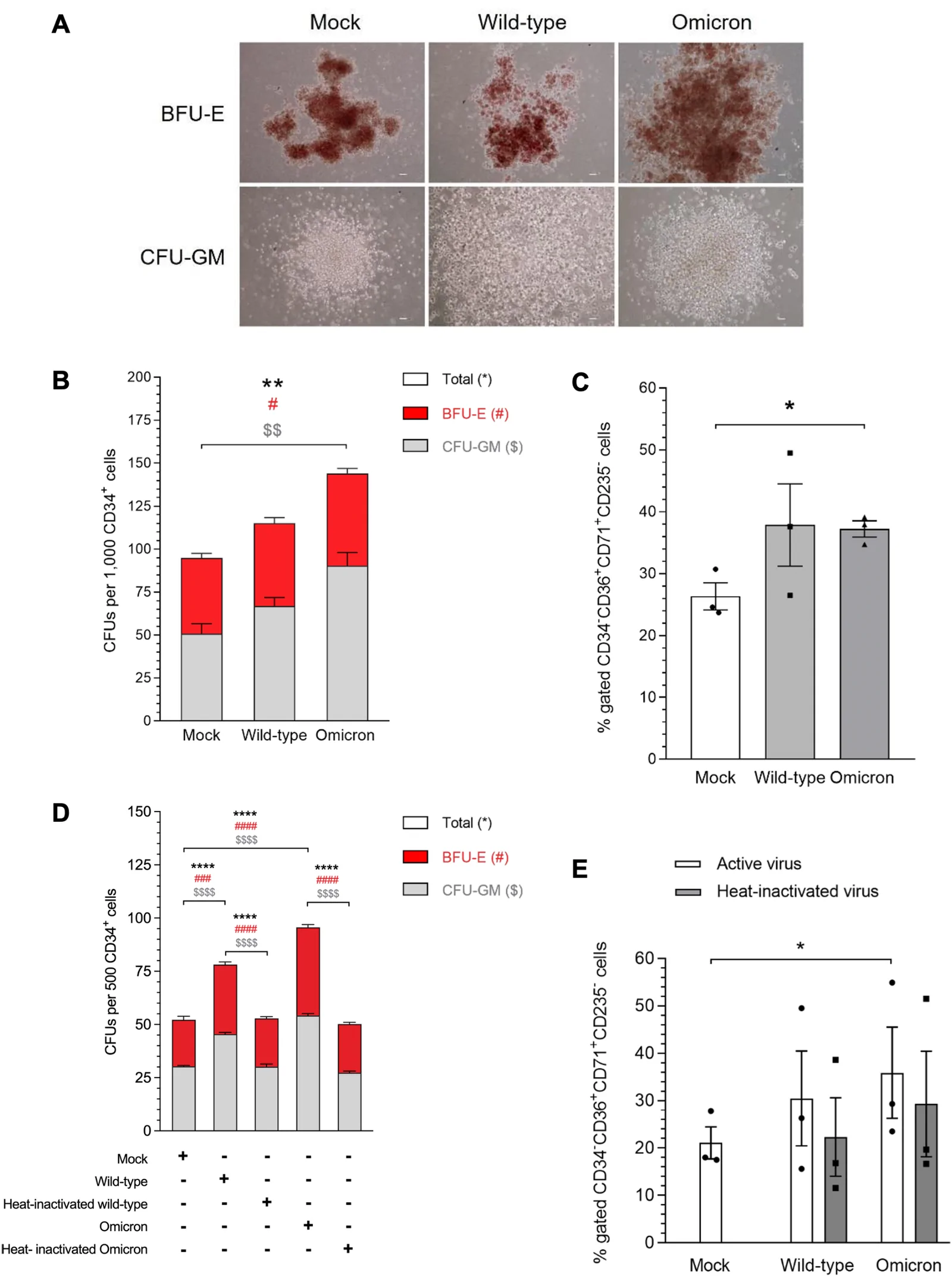
SARS-CoV-2 exposure alters colony-forming output and erythroid progenitor profiles in UCB CD34 ⁺ HSPCs. **(A)** Representative CFU colonies generated from human UCB-CD34^+^ HSPCs after exposure to WT or Omicron SARS-CoV-2 compared to Mock. **(B)** Quantification of burst-forming unit–erythroid (BFU-E), colony-forming unit-granulocyte/macrophage (CFU-GM), and total CFU output **(C)** Frequency of early erythroid progenitors (CD34 ^−^ CD36 ⁺ CD71 ⁺ CD235^−^) following *in vitro* erythroid differentiation of SARS-CoV-2-exposed CD34 ⁺ cells. **(D)** CFU output (BFU-E, CFU-GM, and total) in the virus inhibition experiment comparing active versus heat-inactivated virus. **(E)** Frequency of early erythroid progenitors (CD34 ^−^ CD36 ⁺ CD71 ⁺ CD235^−^) in the virus inhibition experiment. The results represent the means and SEM of three independent experiments. For Fig 5B and 5D, the unpaired t-test were performed among each group by comparing of total CFUs, BFU-E, and CFU-GM between Mock vs. WT, Mock vs. Omicron, WT vs. heat-inactivated WT, and Omicron vs. heat-inactivated Omicron. For Fig 5C and 5E, statistical comparisons among Mock, WT, and Omicron in each group were performed using one-way ANOVA followed by Tukey’s multiple-comparisons test. * *P*  <  0.05, ** P  <  0.01, *** *P*  =  0.0001, **** *P*  <  0.0001.

### HSPC functionality and immune response profiling after Omicron exposure

To define transcriptional response associated with Omicron exposure in HSPCs relative to the unexposed control, we profiled RNA from Omicron-exposed versus uninfected CD34 ⁺ HSPCs using targeted NanoString panels (stem cell characterization and host response), followed by differential expression and Gene Ontology (GO) enrichment analyses. In the stem cell panel, Omicron exposure was associated with increased expression of *BMP4*, *ZNF750*, and *COL6A3* and decreased expression of *SYNPO2*. Interestingly, *BMP4* has been implicated in stress-associated erythropoiesis during inflammatory responses [18,19] (Fig 6A, S1 Table). GO enrichment further indicated that, compared with the unexposed-control group, Omicron-exposed HSPCs showed negative regulation of cell cycle and stem cell differentiation (Fig 6B). In the host response panel, Omicron exposure was associated with upregulation of *CXCR1*, *IFNA14/16*, *ZBP1*, and *FCGR3A/B* and downregulation of *IL20*, *PLIN4*, *CTLA4*, and *TLR5* (Fig 6C, S2 Table). GO analysis indicated enrichment of pathways related to chemokine responsiveness, leukocyte migration, and innate immune priming, consistent with a shift toward a stress- and host-response state (Fig 6D). In contrast, downregulated GO terms were enriched for B-cell and antigen receptor–associated signaling and for regulatory programs linked to myeloid and lymphoid differentiation (Fig 6D). Together, this transcriptional change may associate with SAR-CoV2 exposure which can reflect a general response of HSPC in the activation of host-response pathways and remodeling of cell-cycle and differentiation, aligning with the phenotypic and functional shifts observed in the previous assays.

**Fig 6:**
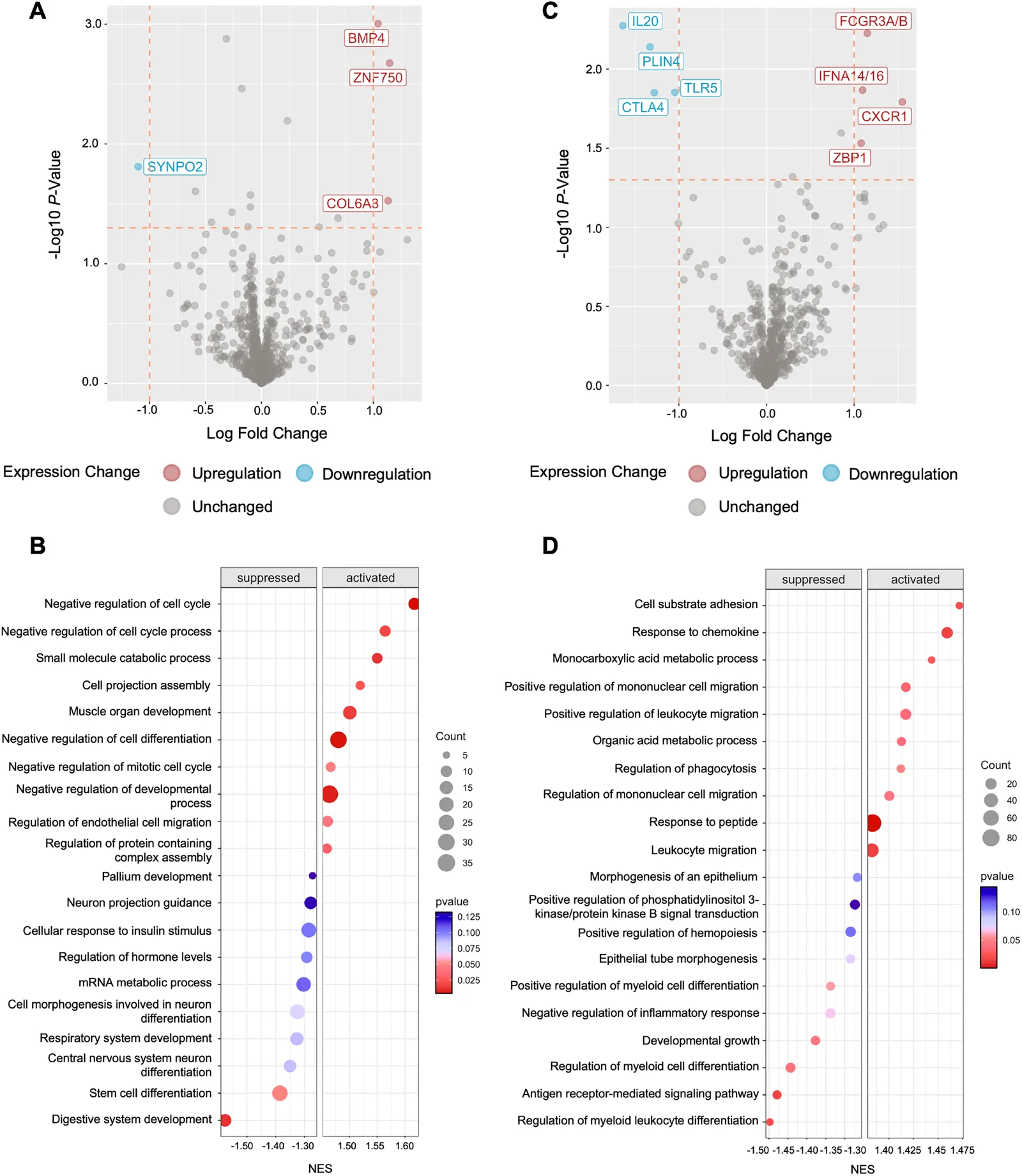
NanoString profiling of Omicron-exposed UCB CD34 ⁺ HSPCs reveals host-response activation and stem/progenitor program remodeling. **(A)** Volcano plot of differentially expressed genes (DEGs) from the Human Stem Cell Characterization panel in Omicron-infected human UCB-CD34^+^ HSPCs compared with uninfected UCB-CD34 + HSPCs at a nominal *P* < 0.05 with |log2 fold change| > 1. **(B)** Gene Ontology (GO) biological process enrichment analysis of DEGs identified from the Human Stem Cell Characterization panel in Omicron-infected human UCB-CD34^+^ HSPCs. **(C)** Volcano plot of DEGs from the Host Response panel in Omicron-infected human UCB-CD34^+^ HSPCs compared with uninfected UCB CD34 ⁺ cells at a nominal *P* < 0.05 with |log_2_ fold change| > 1. **(D)** Gene Ontology (GO) biological process enrichment analysis of DEGs identified from the Host Response panel in Omicron-infected human UCB-CD34^+^ HSPCs.

## Discussion

COVID-19 is widely recognized as a systemic illness, with severe and post-acute disease frequently accompanied by hematologic abnormalities and dysregulated inflammatory signaling [5,20,21]. These observations raise an important unresolved question: beyond indirect effects of systemic inflammation, can early hematopoietic compartments and their stromal niche be directly engaged by SARS-CoV-2 in a variant-dependent manner, with downstream consequences for hematopoietic output. Here, using *in vitro* exposure models, we define variant- and cell type- associated patterns of SARS-CoV-2 NP positivity and remodeling in adult MSCs and UCB CD34 ⁺ HSPCs. Across both systems, positive intracellular NP were observed without detectable infectious virus in supernatants under the conditions tested. These findings indicate that NP positivity provides evidence for the presence of intracellular viral antigen in either MSCs or HSPCs. Therefore, the presence of NP nevertheless coincided with transcriptional and functional remodeling, in line with a recent pseudovirus study showing hematopoietic stress responses can arise without standard evidence of productive infection which directly correlated with our observation [22]. Together with the absence of detectable infectious virus release in our experiment, the detection of NP may reflect restriction at a post-entry stage of the viral life cycle, including viral replication, assembly, or release. However, further assessment of viral replication, including the detection of sub genomic RNAs (sgRNAs) or negative-strand RNA, would be required to confirm intracellular SARS-CoV-2 replication and to determine the specific stage of the viral life cycle at which SARS-CoV-2 is restricted in MSCs and HSPCs.

Our MSC findings highlight why MSC heterogeneity matters in COVID-19–related biology and translation. AD-MSCs showed much higher Omicron-associated NP positivity than WT or Delta, while BM-MSCs showed only modest/variable NP positivity. This suggests that variant effects can add another layer to the well-established idea that MSC tissue source shapes MSC phenotype and function [9,10]. Importantly, SARS-CoV-2 exposure increased *IDO1* in AD-MSCs (strongest with Omicron), indicating activation of an immunomodulatory program even when infectious virus release was undetectable. Because *IDO1* can influence immune tone and niche signaling [23], MSC-immune interactions during infection or recovery may be shaped not only by systemic cytokines but also by virus-triggered activation of niche cells.

These observations are relevant given ongoing interest in MSC-based therapies for COVID-19 and inflammatory lung injury. Meta-analyses and reviews suggest possible benefit signals in severe disease but also emphasize study heterogeneity and the need for larger trials [24,25]. Our study does not assess clinical efficacy or safety; however, the data suggest two practical considerations: (i) MSC tissue source may influence how these cells respond to SARS-CoV-2 exposure and (ii) exposure-associated activation of MSCs may shape their immunomodulatory state, which could influence MSC–immune interactions – an idea that will require direct testing in standard potency assays and *in vivo* models. Future COVID-19 MSC-based therapeutic studies would benefit from more consistent reporting of MSC tissue source, entry-factor profiles, and key immunomodulatory signatures.

Mechanistically, our findings support NRP1 contribute to the increased susceptibility of Omicron exposed AD-MSCs. However, NP positivity was not completely disappeared following NRP1 knockdown. This could be explained by incomplete NRP1 knockdown as showed as the remaining detection of low level NRP1 by immunoblotting and immunofluorescence after siRNA treatment. NRP1 is known to support SARS-CoV-2 entry and infection in other settings, making an entry-related role in MSCs plausible [16]. A broader implication is that variant entry biology may change which “non-canonical” cell types respond to viral exposure. Omicron lineages have been reported to differ from earlier variants in TMPRSS2 dependence and fusogenicity, with entry routes that vary by cell type and protease context [26]. In our system, NRP1-linked processes may help explain the stronger Omicron-associated NP positivity in AD-MSCs. However, our data do not show that NRP1 is sufficient or pinpoint the exact step affected; other attachment factors, endocytic pathways, and post-entry restriction mechanisms may also contribute. Therefore, the NRP1-independent or non-canonical entry pathways may help to explain the persistence of Omicron-associated NP positive. Alternative attachment, entry, or endosomal processing mechanisms, involving factors such as C-type lectin receptors [27,28], the receptor tyrosine kinase AXL [29,30], and cathepsin L [31], may also contribute to the interaction between Omicron and AD-MSCs. Further studies, including NRP1 rescue or receptor-blocking experiments, will be needed to define the specific role of NRP1 and determine whether alternative pathways contribute to the residual NP positivity.

In UCB CD34 ⁺ HSPCs, Omicron exposure was associated with higher NP positivity than WT and enrichment within a phenotype-defined primitive/LT-HSC–like fraction, along with reduced frequencies of primitive fractions. Functionally, Omicron increased clonogenic output (BFU-E and CFU-GM) and promoted accumulation of early erythroid progenitors at day 14, with heat-inactivated controls reducing or eliminating these effects. These findings have several implications. SARS-CoV-2 exposure was associated with alterations in HSPC phenotype and hematopoietic output *in vitro*. They support the idea that primitive hematopoietic compartments can shift into a stress-responsive state after SARS-CoV-2 exposure, consistent with reports of stress hematopoiesis in COVID-19 [32]. Maintenance of low intracellular reactive oxygen species (ROS) level is a feature of quiescent HSCs for preserving their self-renewal and long-term repopulation capacity [33]. Increasing of ROS levels can promote HSC exit from quiescence and differentiation, whereas excessive or prolonged oxidative stress led to impair HSC function [34]. Our results can be implicated that SARS-CoV-2 exposure may induce a stress-responsive state in HSPCs that promotes their lineage differentiation *in vitro*. However, to clarify whether viral internalization contributes to this HSPC function alteration, 4°C adsorption control would be incorporated to distinguish the effect of surface bound viral particles. Moreover, these purposed mechanisms remain to be experimentally validated in physiologically relevant models and patient-derived samples.

Therefore, the transcriptional profiling showed activation of host-response pathways and altered cell-cycle/differentiation programs, providing a molecular correlate for the phenotypic and functional shifts observed after SAR-CoV2 exposure. Together, these data support a testable framework in which SARS-CoV-2 exposure does not need to be productive in HSPCs to alter their state and downstream differentiation trajectories.

Several limitations should be noted. Our study uses *in vitro* exposure models with modest donor/replicate numbers and does not directly measure intracellular replication dynamics. Importantly, the present experiments cannot determine the specific stage at which the viral life cycle is restricted. The detection of intracellular nucleocapsid protein following SARS-CoV-2 exposure with the absence of detectable infectious virus release could reflect restriction at a later stage of the viral life cycle, including replication, assembly, or release. NP positivity should be interpreted as exposure-associated engagement rather than proof of productive infection. “No detectable infectious virus” also depends on assay sensitivity and the sampled time points.

In addition, the “LT-HSC” fraction we studied is immunophenotype-based and not validated by long-term engraftment. Moreover, for AD-MSCs, it should be noted that donor-related factors, including age, sex, and underlying health of AD-MSCs sources might directly or indirectly influence in the susceptibility of Omicron. Recent studies have reported the influence of these donor-related factors effect AD-MSC proliferation, differentiation, senescence, inflammatory responses, and immunomodulatory properties [35–37].

Another major limitation is the using of UCB-derived HSPCs. Although UCB CD34^+^ HSPCs are widely used in research and clinical experiment by retaining the two fundamental properties that define hematopoietic stem cell functions, including self-renewal and multilineage differentiation potential [38,39], the responses observed in UCB-derived HSPCs may not fully reproduce those of adult bone marrow HSPCs exposed to the complex inflammatory environment associated with COVID-19. Finally, we did not model stromal–hematopoietic interactions in an integrated niche system; co-culture and cytokine-conditioned models may better reflect *in vivo* environments for determining how direct viral exposure and inflammation-mediated niche alterations collectively influence hematopoiesis during SARS-CoV-2 infection.

In summary, Omicron exposure was associated with heightened NP positivity in AD-MSCs in an NRP1-linked manner and induced IDO1 expression, while infectious virus release remained undetectable in MSC supernatants under the tested conditions. In UCB CD34 ⁺ HSPCs, Omicron exposure was associated with enriched NP positivity in phenotype-defined primitive compartments, reduced primitive fraction frequencies, increased CFC output, erythroid developmental shifts, and host-response transcriptional remodeling. Together, these findings suggest that variant-specific entry biology can broaden the range of stromal and early hematopoietic cells that respond to SARS-CoV-2 exposure, with potential implications for niche immunoregulation and stress hematopoiesis that warrant further *ex vivo* and *in vivo* validation.

## Supporting information

S1 Table List of NanoString gene probes included in the Stem Cell Panel. (XLSX)

S2 Table List of NanoString gene probes included in the Host Response Panel. (CSV)

S1_raw_images Uncropped Blot Images. (PDF)
