# Supplementary material for: Omicron SARS-CoV-2 shows NRP1-linked susceptibility in adipose-derived MSCs and alters primitive cord blood CD34⁺ HSPCs
Source: PLoS One. 2026 Oct 12;21(10):e0360439. doi: 10.1371/journal.pone.0360439 (PMC13657315; doi:10.1371/journal.pone.0360439)
Supplement: Supplementary file 1 [file pone.0360439.s003.pdf]

Figure 2A

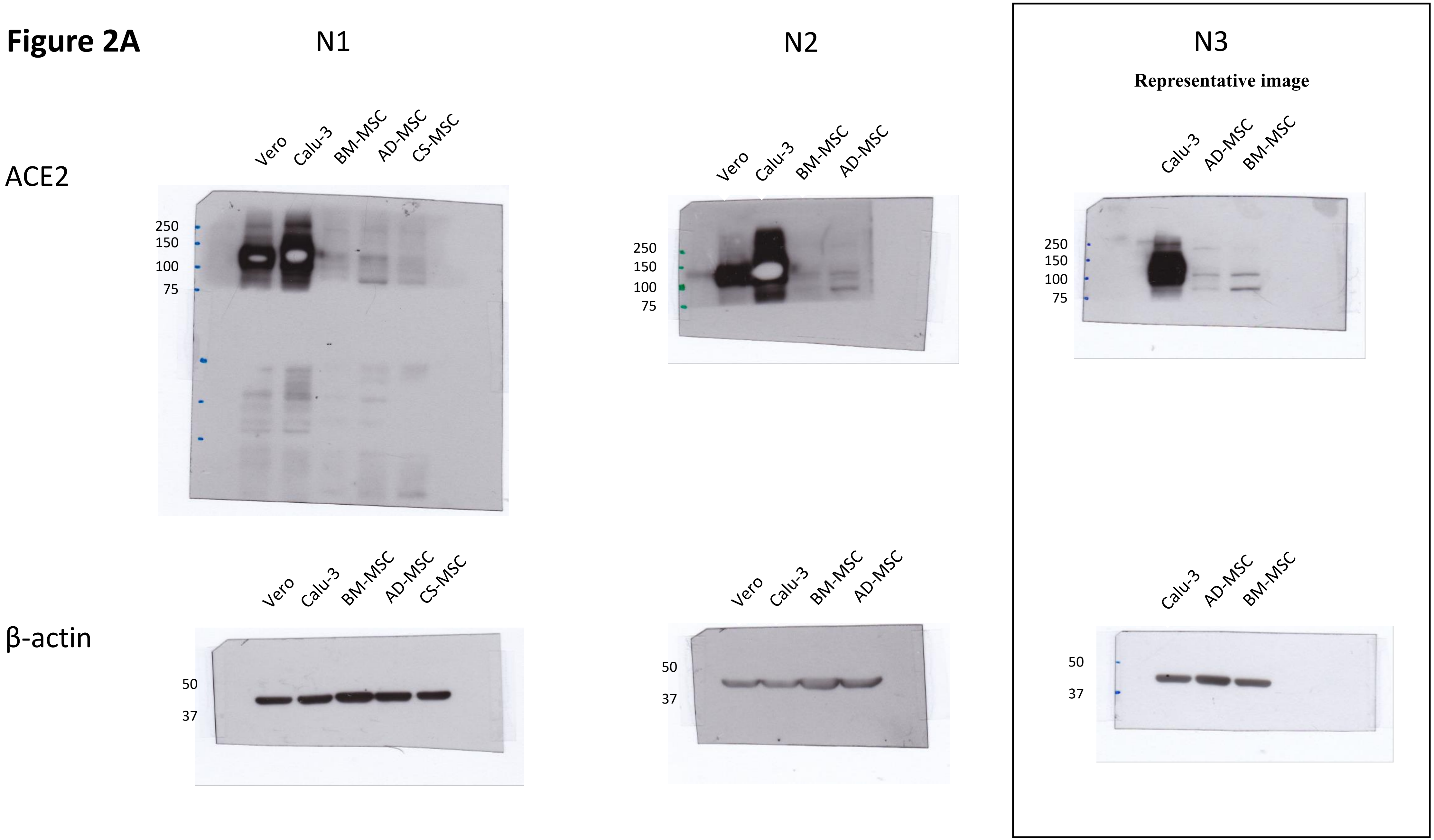
N1
N2
N3
Representative image
ACE2
β-actin
Vero
Calu-3
BM-MSC
AD-MSC
CS-MSC
250
150
100
75
50
37

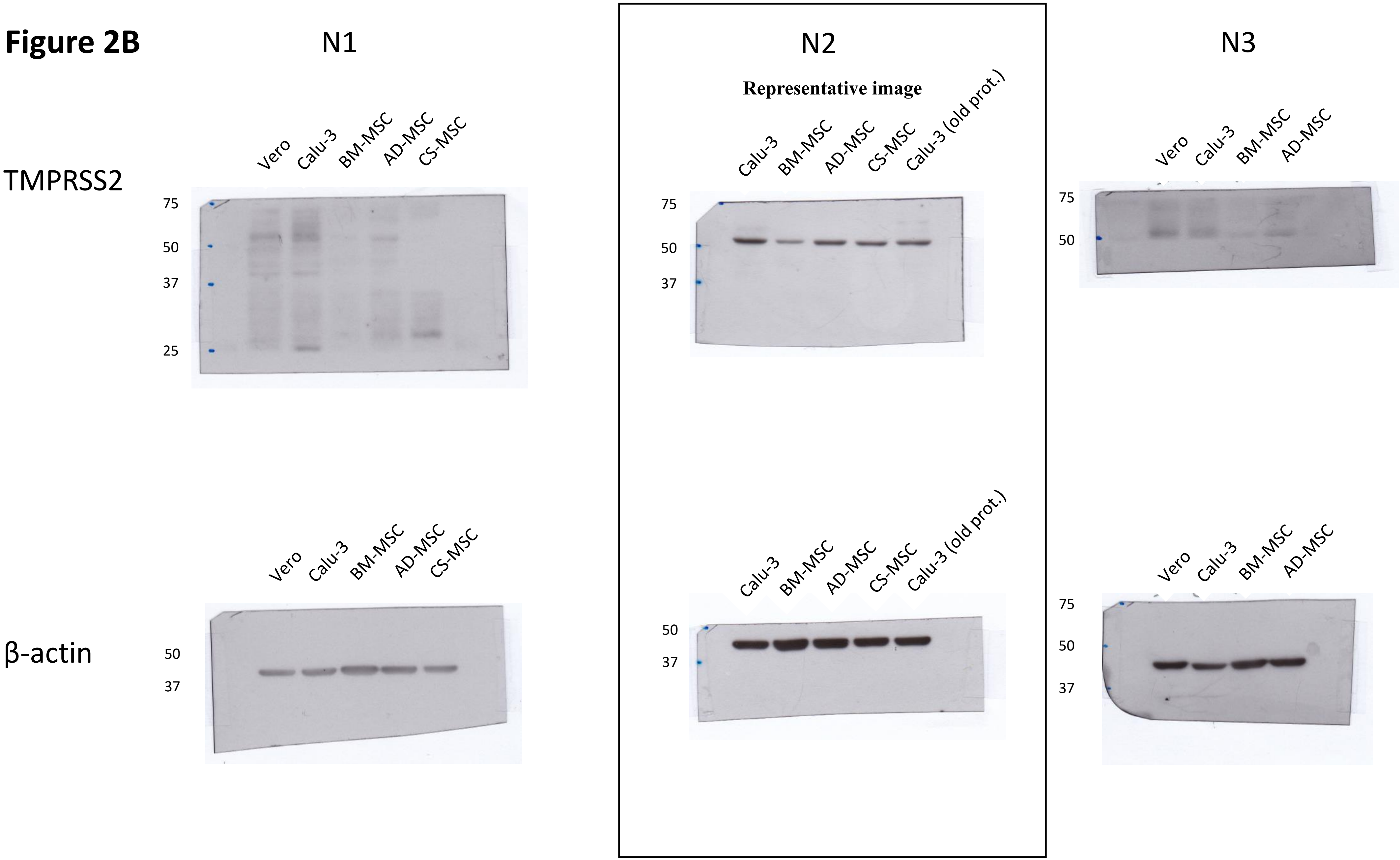

Figure 2B
N1
N2
N3
Representative image
TMPRSS2
β-actin
Vero
Calu-3
BM-MSC
AD-MSC
CS-MSC
Calu-3 (old prot.)
75
50
37
25

Figure 2C

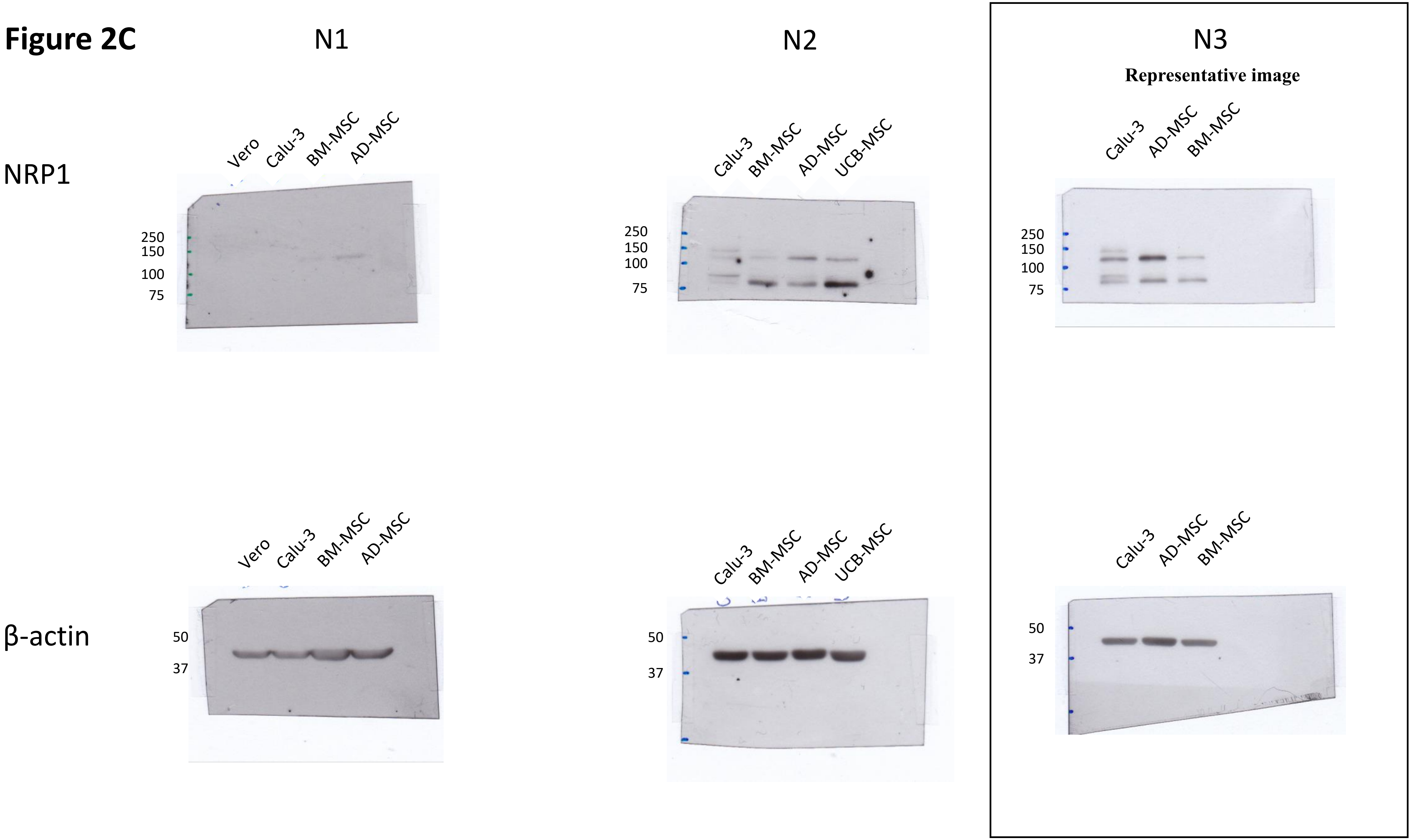
N1
N2
N3
Representative image
NRP1
β-actin
Vero
Calu-3
BM-MSC
AD-MSC
UCB-MSC
250
150
100
75
50
37

Figure 3A

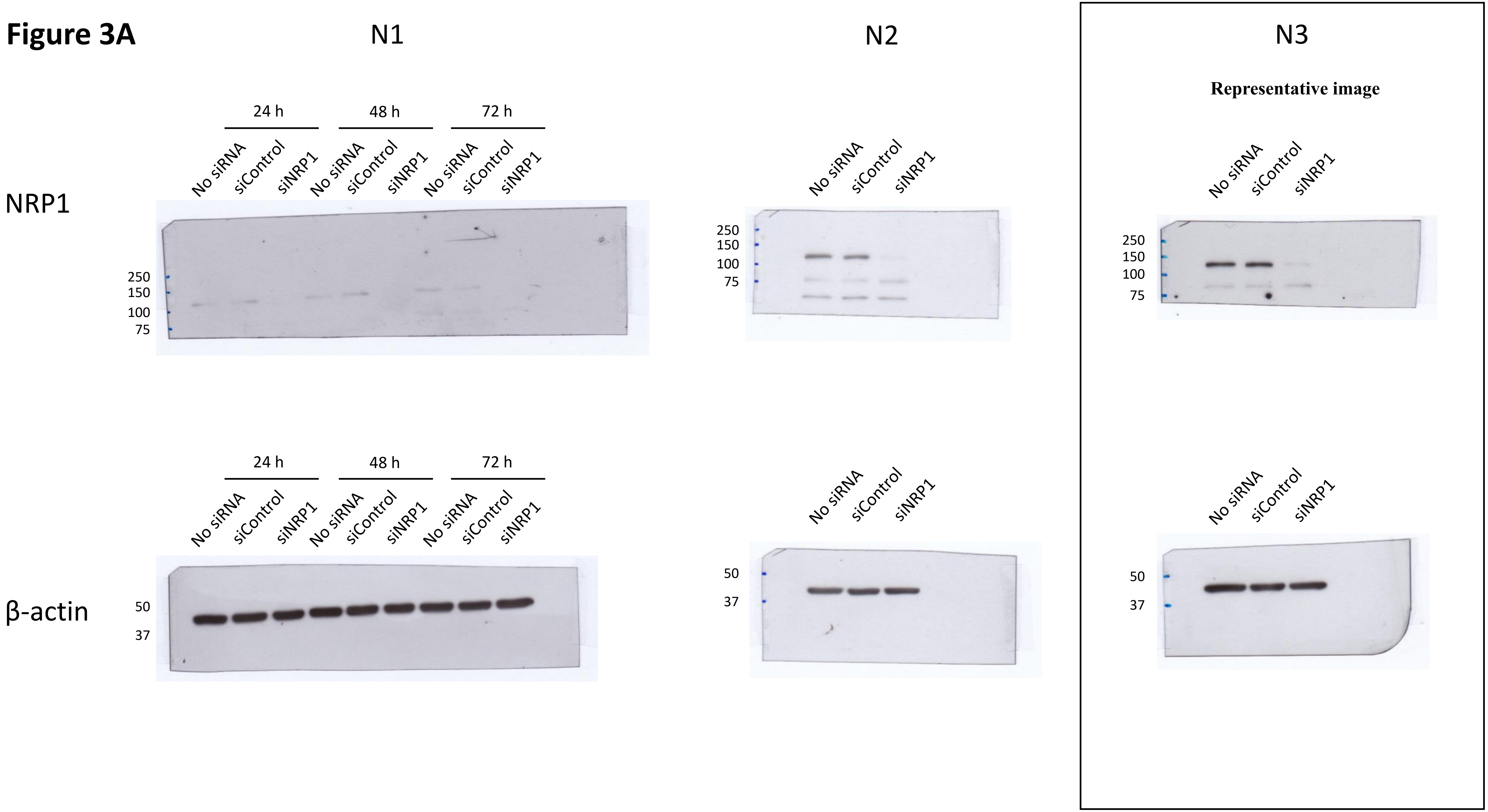
N1
N2
N3
Representative image
NRP1
β-actin
24 h
48 h
72 h
No siRNA
siControl
siNRP1
250
150
100
75
50
37
